# High heterogeneity in the size distribution of the micellar fraction from *in vitro* digestions: sample preparation and reporting recommendations

**DOI:** 10.1002/jsfa.14109

**Published:** 2025-01-07

**Authors:** Roman Will, Claudia Rein, Jan Frank, Johanita Malan

**Affiliations:** ^1^ Department of Food Biofunctionality University of Hohenheim Stuttgart Germany; ^2^ Department of Food Technology Fulda University of Applied Sciences Fulda Germany

**Keywords:** dynamic light scattering, micellar size, *in vitro* digestion, bioactives, emulsion, nanoparticles

## Abstract

**BACKGROUND:**

Understanding the size and surface charge (*ζ*‐potential) of particles in the mixed micellar fraction produced by *in vitro* digestion is crucial to understand their cellular absorption and transport. The inconsistent presentation of micellar size data, often limited to average particle diameter, makes comparison of studies difficult. The present study aimed to assess different size data representations (mean particle diameter, relative intensity‐ or volume‐weighted size distribution) to better understand physiological mixed micelle characteristics and to provide recommendations for size reporting and sample handling.

**RESULTS:**

Dietary compounds (RRR‐α‐tocopherol, retinyl‐palmitate, β‐carotene, curcumin and naringenin) underwent a simplified *in vitro* digestion, whereas foods (spinach and red cabbage) were subjected to both a simplified and the INFOGEST 2.0 digestions. Dynamic light scattering was used to measure size and surface charge of the mixed micelles. A significant percentage of particles above the 200 nm filter cut‐off was observed, indicating aggregation and dynamic size changes in the mixed micellar fraction. Freezing of the mixed micelles notably enhanced the aggregation.

**CONCLUSION:**

The determination of particle size in polydisperse mixed micellar fractions is challenging, and relying solely on average particle diameter can be misleading. Especially in more polydisperse samples, parameters such as polydispersity index and volume‐weighted distribution should accompany average particle diameter data. To minimize the effect of freezing on particle size, we recommend filtering the digesta after storage (freezing), as this leads to similar size distribution compared to mixed micellar fraction measured directly after digestion. © 2025 The Author(s). *Journal of the Science of Food and Agriculture* published by John Wiley & Sons Ltd on behalf of Society of Chemical Industry.

## INTRODUCTION

*In vivo* research investigating the bioavailability of compounds is costly and time consuming; therefore, there is currently much focus on the *in vitro* bioaccessibility of dietary compounds, in particular the lipid‐soluble vitamins and (poly)phenols. Bioaccessibility is normally measured as the fraction of a compound recovered from an *in vitro* digestion after centrifugation and filtration (> 200 nm). As far as lipid‐soluble compounds are concerned, it estimates how much of a compound is released from the consumed matrix, incorporated into mixed micelles and available for intestinal absorption.^1^, ^2^ Not only can *in vitro* bioaccessibility data be used to predict *in vivo* bioavailability of bioactives,^3^, ^4^, ^5^ but also it provides the opportunity to further investigate the mechanisms behind bioavailability modulation.^6^ For example, the role of bioavailability enhancers or inhibitors on the liberation of compounds from the food matrix, their digestive stability, solubility and micellization efficiency, can be determined. Additionally, it is possible to characterize the size and surface charge of the mixed micelles or, generally speaking, particles in the mixed micellar fraction produced during the *in vitro* digestion. Because the micellar size and surface charge (*ζ*‐potential) can affect the cellular absorption of mixed micelles,^7^, ^8^, ^9^ it provides valuable information to better understand micellar absorption. Although there is a sizable amount of research evaluating the size and charge of *in vitro* produced physiological mixed micelles,^10^, ^11^ research that systematically evaluates factors influencing size of mixed micelles, particularly those produced during *in vitro* digestion, is severely lacking. However, where mixed micelles are formed by mixing different components, the size and surface charge of mixed micelles can be affected by the composition of the micelles, including the type of bile acids and concentration of free fatty acids.^12^, ^13^, ^14^ Confirmation of this for *in vitro/in vivo* systems is still required.

Data on these characteristics of physiological mixed micelles produced *in vitro* and measured particularly with dynamic light scattering (DLS) are increasingly being published.^15^, ^16^, ^17^, ^18^ Although DLS is most suitable for monodisperse samples (narrow size distribution), its use is limited for samples containing particles of widely varying sizes, aggregating or light absorbing particles, or samples in complex biological matrices such as intestinal fluids.^19^ Other methods used to characterize mixed micelles, such as NMR spectroscopy and transmission electron microscopy (TEM) have been a dapted andused,^19^, ^20^ but have the disadvantage of low throughput and higher cost, requiring a high level of technological sophistication. NMR spectroscopy and TEM require more complex sample preparation compared to DLS, leading to alteration of natural conditions of the sample. DLS, as a non‐invasive analytical technique, however, is particularly advantageous for studying mixed micelles under near‐native conditions because it minimizes alterations to their structure or behavior, offering insights into their size distribution and stability with minimal perturbation to the sample. Additionally, the majority of studies characterizing the size and charge of mixed micelles use DLS because of its advantages of high throughput, minimal sample preparation, low cost and time‐efficient measurement.

However, micellar size is inconsistently presented as either mean particle diameter, relative volume‐ or intensity‐weighted size distribution. There are also no data on the impact of storage conditions on particle size and surface charge. After *in vitro* digestion, the mixed micellar fraction (centrifuged and filtered digest) of the final digesta is often frozen before further analyses, such as cellular uptake and HPLC analyses, as well as for micellar characterization. General guidelines on the use of DLS for characterization of nanoparticles are available,^19^ but there are no guidelines for the reporting of DLS data for characterization of mixed micelles.

Therefore, the the present study aimed (i) to evaluate the application of DLS data to characterize particles in the *in vitro* mixed micellar fraction using a diverse range of compounds and food matrices; (ii) to provide guidelines for the reporting of the data; and (iii) to propose the best practice for measuring the size and surface charge most accurately, in particularly after storage.

## MATERIALS AND METHODS

### Materials

The compounds digested were RRR‐α‐tocopherol (purity 970 g kg^−1^; DSM Nutritional Products, Basel, Switzerland), retinyl palmitate (1.7 × 10^6^ IU g^−1^; AQUANOVA, Darmstadt, Germany), curcumin (purity 950 g kg^−1^; Jupiter Ley, Okkal, India), naringenin (purity ≥ 980 g kg^−1^; Carl Roth, Karlsruhe, Germany), β‐carotene (purity ≥ 930 g kg^−1^; Sigma‐Aldrich Karlsruhe, Germany) and olive oil (P75343; Sigma‐Aldrich). Fresh spinach and red cabbage were purchased from a local supermarket (Stuttgart, Germany). The selected samples aimed to produce a broad range of *in vitro* mixed micelles, from compounds often found in foods and food products. These included ‘simple micelles’ containing individual fat‐soluble compounds, as well as more ‘complex micelles’, which were produced from a digestion including an oil (crucial for micelle formation) and a whole food matrix. The digestive enzymes and bile used in the *in vitro* digestion, pepsin from porcine gastric mucosa (P7000; ≥ 250 units mg^−1^), pancreatin from porcine pancreas (P7545; 8 × USP specification), porcine bile (B8631) for simplified digestion, bovine bile (B3883) for INFOGEST 2.0 digestion,^21^ and lipase from porcine pancreas type II (L3126) were obtained from Sigma‐Aldrich. The total bile acid assay kit (E‐BC‐K181‐M.96) was purchased from Biomol (Hamburg, Germany). Solutions were prepared using distilled and deionized water (H_2_Odd) (Merck Millipore, Schwalbach, Germany).

### Sample preparation

Fresh spinach and red cabbage were prepared on the day of purchase. Damaged leaves, stems of spinach and outer layers of red cabbage were removed. To remove impurities, spinach and red cabbage were submerged in tap water and soaked for 5 min. This procedure was repeated four‐times using H_2_Odd in the last repetition. The leaves of the spinach and red cabbage were dried in the dark, at room temperature for 2 h. Leaves were freeze‐dried until no weight change occurred (LyoQuest‐85 freeze drier; Azbil Telstar Technologies, Terrassa, Spain), ground using a laboratory mill and stored at −80 °C until *in vitro* digestion.

### Simulated gastrointestinal digestion

For the individual compounds (RRR‐α‐tocopherol, retinyl palmitate, β‐carotene, curcumin and naringenin), 170 μg was digested with or without 250 μL of olive oil. The freeze‐dried foods (100 ± 5 mg) were rehydrated to the initial moisture content (spinach: 896 g kg^−1^ fresh weight; red cabbage: 891 g kg^−1^ fresh weight) with H2Odd before the *in vitro* digestion, with or without 250 μL of olive oil.

The INFOGEST 2.0^21^ and a simplified version previously described by Rodrigues *et al*.^22^ were performed to assess the impact of different *in vitro* digestion protocols on the size outcome. The respective digestion parameters for the two *in vitro* digestions used and enzymatic activity of pepsin, pancreatin and lipase are provided in the Supporting information (Table S1). For the INFOGEST 2.0 protocol, pepsin activity, and lipase activity of porcine pancreatin and lipase were assessed according to protocols suggested by Brodkorb *et al*.^21^ Total bile concentration in bovine bile was quantified with a total bile acid assay kit. The INFOGEST 2.0 method was applied to the rehydrated foods (with and without olive oil), whereas a simplified method was applied to pure substances and foods (with and without olive oil). Although the original INFOGEST 2.0 protocol recommends a gastric pH of 3.0, we adjusted the pH to 2.5 to ensure similar pepsin activity as in the simplified *in vitro* digestion method. For the control digestions, no compound or food was added (volume of compound/food was replaced by 1 mL of H2Odd).

Final digesta were centrifuged (4700 × *g* for 1 h at 4 °C) and the supernatant, representing the soluble fraction, was collected. To obtain the mixed micellar fraction, the soluble fraction was filtered according to recommendation of the standardized INFOGEST 2.0 method with a 200‐nm sterile syringe filter (PES‐FiltropurS; Sarstedt, Nümbrecht, Germany). Each sample was digested in triplicate. Samples not analyzed directly after digestion, were stored at −20 °C to simulate storage conditions.

### Particle size and charge measurements

Size and surface charge (*ζ*‐potential) of the soluble and mixed micellar fraction were measured in a folded capillary zeta cell (Malvern Pananalytical, Malvern, UK) using a Zetasizer® Nano ZSP at 25 °C, 633 nm laser wavelength, 90° scattering angle and 173° for backscattering. The soluble and mixed micellar fraction was analyzed on the day of the *in vitro* digestion (referred to as **filtered**), and an aliquot was frozen (−20 °C) overnight and measured the following day (referred as **filtered‐frozen**). Additional treatment was performed where the soluble fraction (supernatant of the centrifuged digesta) was frozen at −20 °C, and then thawed at room temperature the following day in the dark, and filtered directly before the measurement (referred to as **frozen‐filtered**). All samples were diluted 1:10 with H2Odd and mixed shortly (2–3 s), and size and *ζ*‐potential were measured immediately after filtering. Size and surface charge of each replicate were measured in four consecutive runs. An additional experiment was performed to investigate the effect of the dilution medium [intestinal fluid (SIF) and H2Odd] on the particle size data from the food samples digested with the INFOGEST 2.0. All three samples (filtered, filtered‐frozen and frozen filtered) were diluted 1:10 in SIF and H2Odd before measurement.

The primary particle size data from DLS include the polydispersity index (PDI) and the size distribution based on the intensity of the scattered light. From the intensity data, the mean particle diameter (*z*‐average), volume‐weighted and number‐weighted size distributions are derived, in addition to the individual volume‐weighted peak data and the number‐weighted mean particle diameter.^23^ A graphical presentation of these related parameters is given in the Supporting information (Fig. S1).

*z*‐Average (*D*_*Z*_) is the intensity‐weighted mean hydrodynamic size calculated by the methods of cumulants analysis defined by ISO 13321 and ISO 22412 assuming a single particle size and applying a single exponential fit to the autocorrelation function.^24^, ^25^ The *z*‐average is expressed by the Stokes–Einstein equation^19^, ^26^:

(1) $$D_{Z}=\frac{k_{B}T}{3\pi \eta D_{t,\mathrm{avg}}}$$

where

*D*_*z*_ is the mean hydrodynamic diameter (*z*‐average), *D*_*t,avg*_ is the translational diffusion coefficient (by DLS), *k*_*B*_ is Boltzmann's constant, *T* is the thermodynamic temperature and *η* is the dynamic viscosity.

According to the Rayleigh approximation, the scattering intensity is proportional to the 6th power of the particle radius and is expressed by^27^:

(2) $$\%I_{a}=\frac{a^{6}N_{a}\times 100}{a^{6}N_{a}+b^{6}N_{b}}$$

where

*%I*_*a*_ is the intensity‐weighted distribution of particles with size *a* relative to amount of intensity of particles with size *a*, and *N*_*a*_ and *N*_*b*_ are molecules *N*_*a*_ and *N*_*b*_ of size *a* and *b*, respectively.

The intensity‐weighted distribution can be transformed into a volume‐weighted distribution, reflecting the relative proportions of different particle sizes based on their volume or mass, rather than the intensity of scattered light. This conversion is achieved using Mie theory.^28^ According to the Rayleigh approximation, the mass of spherical particles is proportional to the cube of their size (size^3^)^23^:

(3) $$\%V_{a}=\frac{a^{3}N_{a}\times 100}{a^{3}N_{a}+b^{3}N_{b}}$$

where *%V*_*a*_ is the volume‐weighted distribution of particles with size *a* relative to amount of volume of particles with size *a*, and *N*_*a*_ and *N*_*b*_ are molecules *N*_*a*_ and *N*_*b*_ of size *a* and *b*, respectively.

Intensity‐weighted distribution can be further converted to number‐weighted distribution representing number of particles at a specific size relative to the total amount of particles in solution^23^:

(4) $$\%N_{a}=\frac{{\text{aN}}_{a}\times 100}{{\text{aN}}_{a}+{\mathrm{bN}}_{b}}$$

Where *%N*_*a*_ is the number‐weighted distribution of particles with size *a* relative to amount of particles with size *a*, and *N*_*a*_ and *N*_*b*_ are molecules *N*_*a*_ and *N*_*b*_ of size *a* and *b*, respectively.

### Statistical analyses

The results were analyzed using Prism, version 9.3.1 (GraphPad Software Inc., San Diego, CA, USA). One‐way analysis of variance followed by a Bonferroni's post‐hoc test was used to compare differences between treatments. *P* < 0.05 was considered statistically significant.

## RESULTS

### Presentation of characterization data

#### Estimation of micellar stability

The PDI of the mixed micellar fractions varied between 0.143 and 0.833 (see Supporting information, Table S2). The percentage of particles above the bioaccessibility filter cut‐off (< 200 nm) was 1–60%, although samples were filtered through a 200‐nm membrane directly before DLS measurements (see Supporting information, Table S3). In general, the variation in the size data was high, both within one measurement cycle including four consecutive runs per sample, and between repeated samples (*n* = 3; see Supporting information, Table S4). Shifts in size distribution, within one measurement cycle, towards bigger sizes (Digestion#1), smaller sizes (Digestion#2) and both bigger and smaller sizes (Digestion#3) were observed (see Supporting information, Table S4).

Additional size measurements were done using SIF instead of H2Odd for the dilution of the micellar fraction of digested spinach and red cabbage with and without olive oil (see Supporting information, Fig. S2 and Table S5). The purpose of this approach was to test whether variation in particle size and low stability, indicated by PDI and high percentage of particles larger than 200 nm, resulted from the dilution medium or is an inherent feature of mixed micellar fraction. There was no significant difference between the average PDI of samples diluted in SIF (0.393 ± 0.162) compared to H_2_Odd (0.357 ± 0.151). When diluted in SIF, size‐changes were also observed in the volume‐weighted size distribution during one measurement cycle, including shifts towards bigger and/or smaller sizes, but to a lesser extent than diluted in H_2_Odd (see Supporting information, Table S6). The percentage of particles, when diluted in SIF, above the bioaccessibility filter cut‐off (< 200 nm) was 12.9–71.6%.

#### Transformation of intensity data to volume and number distributions

**Figure 1:**
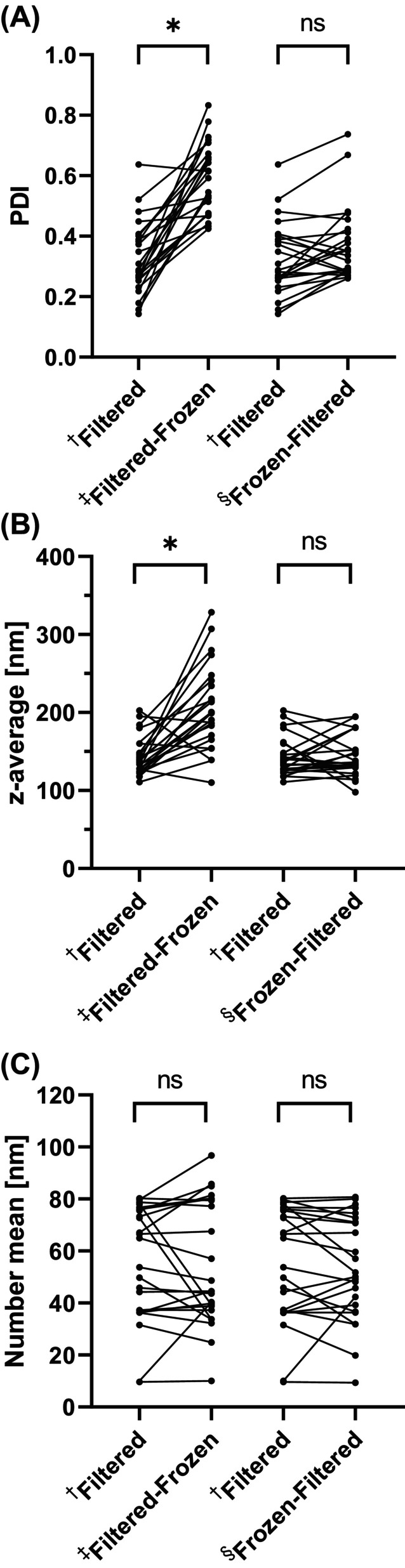
The effects of different combinations of storage (freezing) and filtering (200 nm) on (A) the polydispersity index (PDI), (B) z‐average (mean particle diameter of intensity size distribution) and (C) number mean (mean particle size of number‐weighted size distribution) of *in vitro* mixed micellar fractions. Data are summarized from the Supporting information (Tables S2, S3 and S4). ^†,‡,§^The *in vitro* mixed micellar fractions were measured directly after digestion (^†^filtered) and compared to the same sample (line connections) measured after storage (freezing) (^‡^filtered‐frozen) and to the same sample of which the unfiltered fraction was stored (frozen), followed by filtration directly before the measurement (^§^frozen‐filtered). Each dot (*n* = 24) represents the mean of a sample (*n* = 3). Asterisk indicates significant difference (*P* < 0.05).

**Figure 2:**
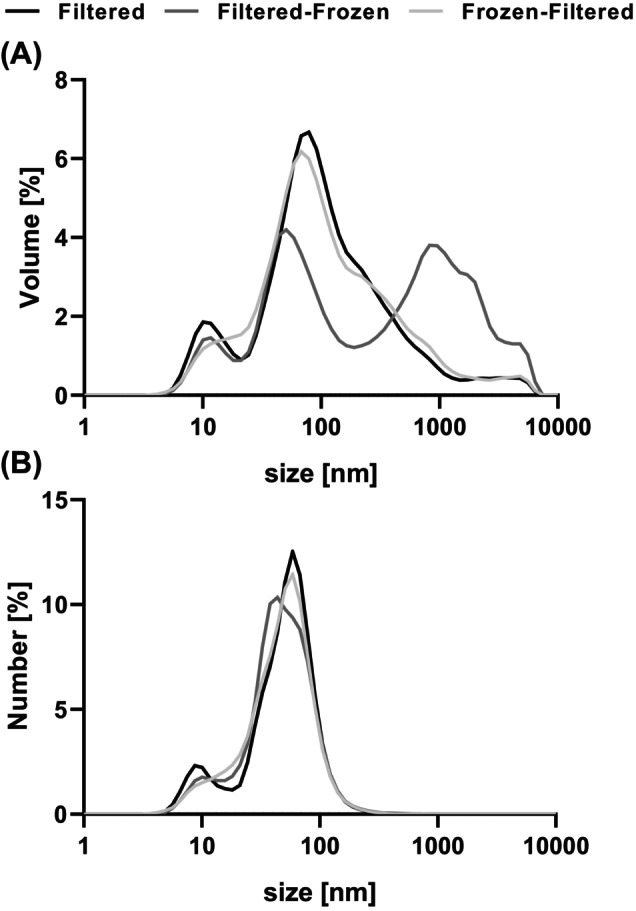
The effects of different combinations of storage (freezing) and filtering (200 nm) on (A) the volume‐weighted^†^ and (B) number‐weighted^†^ size distributions of the *in vitro* mixed micellar fraction. Samples were measured directly after digestion (filtered), after storage (freezing) (filtered‐frozen) or after storage (freezing) of the unfiltered fraction, followed by filtration directly before the measurement (frozen‐filtered). ^†^Volume or number of particles at a specific size, as percentage of the total volume or number of particles, respectively. Distributions represent the mean of all *in vitro* digestions performed (*n* = 24) and are depicted on a logarithmic scale.

Intensity distribution is the raw form of light scattering data. Volume‐weighted and number‐weighted size distributions are the volume or number of particles at a specific size, as percentage of the total volume or number of particles, respectively. Volume‐ and number‐weighted size distributions are derived from the intensity data. Samples displayed here represent the range of different size distributions observed from our larger data set (Figs 1 and 2).

**Figure 3:**
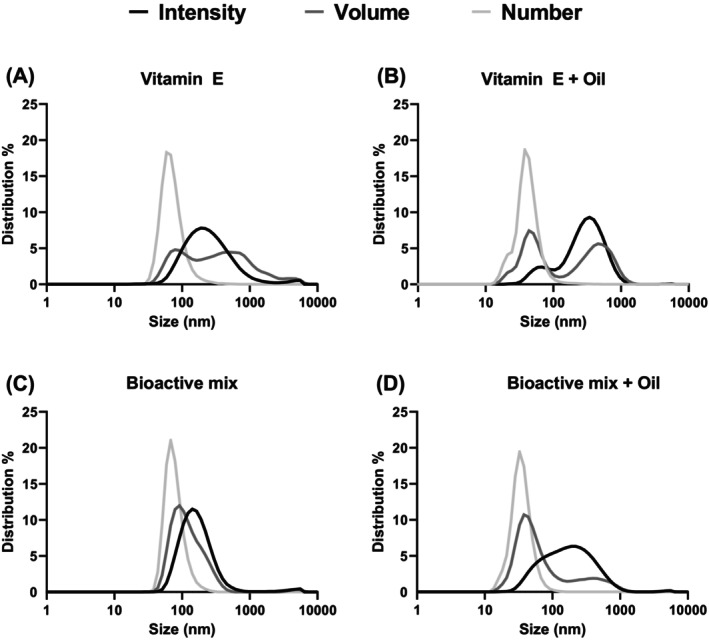
Size distributions (nm) of the *in vitro* mixed micellar fraction of (A and B) vitamin E and (C and D) a compound mix^†^, digested with or without olive oil. Intensity distribution (black graph) is the primary data form, and derived from this, are the volume‐weighted (dark grey graph) and number‐weighted (light grey graph) size distributions representing the volume or number of particles at a specific size, as percentage of the total volume or number of particles, respectively. Data are depicted as means on a logarithmic scale (*n* ≥ 8). ^†^Vitamin E, vitamin A, β‐carotene, curcumin and naringenin.

Broader intensity‐weighted distributions, differing substantially from the volume distributions, were observed in the more polydisperse samples (PDI > 0.3) (Fig. 3A,B,D), compared to the more monodispersed sample (Fig. 3C). This is important because the PDI of almost half of the mixed micellar fractions of the total dataset was larger than 0.3 (see Supporting information, Table S2). By contrast to the intensity data, peaks at smaller sizes were observed in the volume distribution data of polydisperse samples (Fig. 3A,B,D). The number distributions (light grey graph) differed the most from the intensity distributions, yielding singular peaks, representing a large number of small micelles. Intensity, volume and number distributions from the mixed micellar fractions diluted in SIF compared to H_2_Odd were similar, especially the intensity distribution (see Supporting information, Fig. S2). Although the volume‐ and number‐weighted size distributions were relatively broad for H2Odd and SIF, a slightly higher percentage of smaller particles (10–100 nm) was observed in the samples diluted in SIF.

The z‐average (Table 1; see also Supporting information, Fig. S1A) is the scattering intensity‐weighted mean particle diameter, calculated by the Zetasizer® software from the intensity distribution, with the crucial assumption of a narrow size distribution. From the volume distribution curves, each volume‐weighted peak can be characterized by calculating the area under the curve (AUC) of each peak relative to the total AUC given in percentage (Table 2; see also Supporting information, Fig. S1B). For the number‐weighted distribution, the number‐weighted average particle diameter can be calculated (see Supporting information, Fig. S1C).

**Table 1:** Polydispersity index (PDI), *z*‐average (mean particle diameter of intensity size distribution)^a^ and number mean (mean particle size of number‐weighted size distribution)^a^ of the *in vitro* mixed micellar fraction of vitamin E and a compound mix^b^ digested with or without olive oil

	PDI	*z*‐average (nm)	Number mean (nm)
Vitamin E	0.389 ± 0.011	184.3 ± 11.4	76.1 ± 12.2
Vitamin E + olive oil	0.401 ± 0.077	202.6 ± 28.3	44.2 ± 14.6
Compound mix^2^	0.218 ± 0.015	127.3 ± 1.6	79.8 ± 6.4
Compound mix^2^ + olive oil	0.407 ± 0.060	126.8 ± 11.1	36.2 ± 6.9

*Note*: Data are as the mean ± SD (*n* ≥ 8).

^a^ See Supporting information (Fig. S2).

^b^ Vitamin E, vitamin A, β‐carotene, curcumin and naringenin.

**Table 2:** Mean particle size of individual volume‐weighted peaks and the area under the curve (AUC) of each peak, relative to the total AUC (%)^a^ of the *in vitro* mixed micellar fraction of vitamin E and a compound mix^b^ digested with or without olive oil

	Peak 1 nm (%)	Peak 2 nm (%)	Peak 3 nm (%)
Vitamin E	102.9 ± 25.7 (31.8)	632.9 ± 292.9 (64.0)	4128.4 ± 366.0 (4.1)
Vitamin E + olive oil	55.4 ± 9.7 (46.8)	493.3 ± 75.3 (53.1)	4987.0 ± 0.0 (0.1)
Compound mix^2^	132.4 ± 4.4 (98.7)	4749.3 ± 117.1 (1.3)	‐
Compound mix^2^ + olive oil	56.4 ± 10.9 (80.6)	456.1 ± 82.8 (18.9)	4519.5 ± 262.4 (0.6)

*Note*: Data are the mean ± SD (*n* ≥ 8). In brackets, percentage AUC of each peak to total AUC.

^a^ See Supporting information (Fig. S2).

^b^ Vitamin E, vitamin A, β‐carotene, curcumin and naringenin.

The single volume‐weighted peak of the more monodisperse (PDI = 0.218) compound mix without olive oil was 132.4 nm (Table 2), which is very similar to the mean particle diameter (127.3 nm), whereas the number mean was 79.8 nm (Table 1). However, in samples with high polydispersity, there were substantial differences between the mean particle diameter, number mean (Table 1), and individual volume‐weighted peaks (Table 2). The individual volume‐weighted peaks of vitamin E were 102.9, 632.9 and 4128.4 nm, and thus 0.6‐, 3.4‐ and 22.4‐times larger than the mean particle diameter (184.3 nm), respectively, whereas the number mean was 76.1 nm. The individual volume‐weighted peaks of vitamin E + olive oil were 55.4 and 493.3 nm and 0.3‐ and 2.4‐times larger than the mean particle diameter (202.6 nm), whereas the number mean was 44.2 nm. The addition of olive oil to the compound mix resulted in a similar mean particle diameter (126.8 nm), but volume‐weighted peak sizes were 56.4 and 456.1 nm and the number mean notably lower (36.2 nm). The mean particle diameter (204.2 ± 147.4 nm) and the number mean (70.0 ± 57.4 nm) of mixed micellar fraction diluted in SIF did not significantly differ from the mean particle diameter (188.2 ± 53.1 nm) and the number mean (65.4 ± 42.5 nm) diluted in H2Odd.

#### Surface charge

Unlike size data, the surface charge, expressed as *ζ*‐potential, was a more robust parameter, as for all samples the variations were low (see Supporting information, Figs S3 and S4). *ζ*‐potential of particles in the mixed micellar fraction of digested whole foods without olive oil ranged between −21.1 and −37.7 mV, and, for individual compounds, between −59.2 and −65.2 mV, which is comparable to empty digestions (whole food: −23.4 to −47.2 mV; individual compounds: −59.2 mV). Addition of olive oil decreased *ζ*‐potential for whole foods by −9.2 to 35.1 mV, and, for individual compounds, by −19.8 to −27.9 mV, representing the difference between empty digestion and digestion of olive oil (−21.6 mV) (see Supporting information, Fig. S3).

### Effect of storage on mixed micellar size and surface charge

#### PDI

The particle size and surface charge of the mixed micellar fraction were measured directly after completing the digestion, centrifuging, and filtering (in text referred to as filtered) and was used as the control. To investigate the effect of storage on these characteristics, the mixed micellar fraction was frozen at −20 °C overnight (referred to as filtered‐frozen). The PDI (see Supporting information, Table S2), mean particle diameter (see Supporting information, Table S7) and number mean (see Supporting information, Table S8) data were then compared (Fig. 1). Because notable changes in size distribution were observed after freezing, we evaluated an additional treatment: Instead of freezing the filtered supernatant (filtered‐frozen), the supernatant was frozen and then filtered directly before measurement (referred to as frozen‐filtered).

The heterogeneity of the mixed micellar fraction increased significantly after freezing: PDI for filtered samples ranged from 0.143 to 0.637, while filtered‐frozen samples ranged from 0.425 to 0.833 (Fig. 1; see also Supporting information, Table S2). On average, freezing increased the PDI by 0.267 ± 0.163. The increase in PDI was accompanied by a broader volume‐weighted distribution and the presence of additional larger particles (Fig. 2, dark grey graph). The PDI of samples filtered after freezing (frozen‐filtered) ranged from 0.260 to 0.737 and did not differ significantly from the control (filtered) (Fig. 1; see also Supporting information, Table S2). A similar trend was observed in the samples diluted in SIF, where more filtered‐frozen samples differed significantly from the filtered samples, compared to the alternatively treated frozen‐filtered samples (see Supporting information, Table S5).

#### Mean particle diameter

The mean particle diameter ranged from 110.7 to 202.6 nm for filtered samples, 110.0 to 328.4 nm for filtered‐frozen samples and 97.9 to 194.7 nm for frozen‐filtered samples (Fig. 1; see also Supporting information, Table S7). Freezing after filtration significantly increased particle diameter in 75% of the tested samples by 37.0 to 192.2 nm. The mean particle diameter of the frozen‐filtered samples was not significantly different from the control (filtered). For samples diluted in SIF, there was overall, no significant difference in the mean particle diameter data (see Supporting information, Table S5) and the intensity curves compared to dilution in H2Odd (see Supporting information, Fig. S2).

#### Volume‐weighted distribution

In the volume‐weighted distribution, the majority of the particles of the filtered (control) and frozen‐filtered samples was around 80 nm. In the filtered‐frozen samples, this peak was reduced and an additional peak was observed at a larger particle size of 850 nm (Fig. 2). In line with this, the percentage of particles above the filtering cut‐off (> 200 nm) was 1–60% for filtered and 2–62% for frozen‐filtered, but increased to 5–86% after freezing of the filtered samples (filtered‐frozen) (Fig. 2; see also Supporting information, Table S3). When diluted in SIF, the size distribution of the frozen‐filtered samples, was also similar to that of the filtered samples, whereas freezing (filtered‐frozen) reduced the percentage of small particles (< 100 nm) (see Supporting information, Fig. S5).

#### Surface charge

Overnight freezing at −20 °C did not affect the surface charge of the particles in the mixed micellar fraction from *in vitro* digestion neither before, nor after filtering (filtered‐frozen and frozen‐filtered) (see Supporting information, Figs [Link], [Link] and S6).

## DISCUSSION

### Presentation of characterization data

#### Characteristics of particles in *in vitro* mixed micellar fractions

Generally, data published on the soluble or micellar fractions from *in vitro* digestions, include either the *z*‐average (intensity‐weighted mean particle diameter in nm) or the percentage volume distribution. To accurately characterize size of mixed micelles from *in vitro* digestions and to provide guidelines for the size reporting, all available parameters were evaluated (Fig. 3 and Tables 1 and 2). From the intensity, volume and number distribution, it appears that in the *in vitro* mixed micellar fraction, relatively small numbers of big particles contributed strongly to intensity distribution and, to a lesser extent, to volume distribution.

PDI, a measure of the particle size heterogeneity in colloidal solutions, can range between 0 and 1, where values ≤ 0.1 are considered highly monodisperse, 0.1–0.4 moderately polydisperse and ≥ 0.4 highly polydisperse.^19^ Monodisperse solutions have minimal variation in particle size. By contrast, polydisperse solutions contain particles of multiple sizes, which could be a result of fusion, aggregation or agglomeration of smaller particles during preparation, storage, or analysis. In solutions with high polydispersity (PDI > 0.3) or more than one peak, mean particle diameter is less reliable, and thus, less suitable to characterize particle sizes.^19^

The moderate to high PDI values of our data (0.143–0.637) suggested strong dynamics of mixed micelles produced during *in vitro* digestion (see Supporting information, Table S2). The high percentage of particles larger than 200 nm (after 200 nm filtering) implies that the particles in the mixed micellar fraction had low stability, resulting in changes in particle size within minutes after filtration. This might have been because mixed micelles are not solid or rigid particles, but undergo dynamic size changes due to molecular interactions.^12^, ^29^, ^30^ By contrast, artificially produced micelles consisting of various lipid‐soluble (poly)phenols emulsified using polysorbate‐80 showed lower size variation compared to mixed micelles analyzed in our study.^31^ However, after these polysorbate‐80 micelles were subjected to *in vitro* digestion, the size range of particles increased notably.^31^ This observation indicates that mixed micelles produced by *in vitro* digestion are less stable and undergo stronger dynamical size changes compared to artificially produced micelles. Because mixed micelles are formed by the assembly of amphiphilic molecules, these processes might continue after completion of the *in vitro* digestion, resulting in shifts in the size distribution. The exact interactions are difficult to predict and probably highly complex because mixed micelles are composed of varying concentrations of different components, such as bile salts, phospholipids and cholesterol,^32^, ^33^ which micellize different fatty acids and lipid‐soluble compounds.

Among other, factors such as pH, temperature, bile concentration and ionic strength (the charge and concentration of ions in a solution) are critical for micelle stability.^30^, ^34^, ^35^ It was possible that the micellar instability was enhanced by different ionic strengths between mixed micellar fraction (mixture of simulated digestive fluids) and dilution medium of H_2_Odd. This might have caused electrostatic interactions, resulting in a disruption of the hydrophilic surface and destabilization of the particles in the mixed micellar fraction. Various studies have reported that ionic strength affects the size of mixed micelles formed from bile salts.^34^, ^36^, ^37^ Because of the high polydispersity we re‐tested digested foods (with and without olive oil) comparing dilution in distilled and deionized water and SIF (see Supporting information, Fig. S2 and Tables S5 and S6). We found no substantial difference regarding micellar stability and size distributions. Minor differences that were observed cannot with certainty be attributed to the dilution medium as overall high variations appeared in water and SIF.

### Limitations of intensity derived size data

In the more polydisperse samples, broader intensity‐weighted distributions were observed, contrasting significantly with the volume distributions, as seen in Fig. 3(A,B,D). By contrast to the intensity data, the volume distribution data for polydisperse samples revealed peaks at smaller sizes. Specifically, the number distributions exhibited the most pronounced differences from the intensity distributions, representing a large number of small micelles.

The intensity distribution is the raw form of light scattering data and shows the intensity with which light is scattered by the particles in a solution (Fig. 3, black graph). Most intensity distribution curves had a single peak, differing in width, with a smaller peak at 10–100 nm, observed in some cases (Fig. 3B). The scattering intensity is proportional to the sixth power of the particle radius^38^: a particle with a diameter of 50 nm would result in light scattering intensity one million times higher than a particle with a diameter of 5 nm (see Supporting information, Fig. S7). As a result of the overrepresentation of larger particles, intensity data are useful to detect aggregation in monodisperse solutions because slight particle size increases would strongly amplify the intensity signal of these larger particles. However, for the characterization of the relatively polydisperse mixed micellar fractions, as found in the present study, scattering intensity data are of limited use.

Volume‐weighted and number‐weighted size distributions are the volume or number of particles at a specific size, as a percentage of the total volume or number of particles, respectively (Fig. 3; see also Supporting information, Fig. S2). These curves are derived from the intensity data through the application of mathematical equations utilizing Mie theorem, which requires the optical properties (refractive index) and assumes that all particles are spherical and homogenous.^19^ If all particles are spherical and of similar size, all three representations (intensity, volume, number) should be similar. However, this was not the case for the most transformations from intensity to volume‐weighted size distributions, especially for polydisperse samples (Fig. 3A,B,D). Therefore, given the polydisperse nature of numerous samples, transforming from intensity to volume is preferred to assess the impact of larger particles on the overall size variability.

The polydisperse size distribution (high PDI) of the mixed micelle fraction limits the use of DLS which works the best for monodisperse samples because of the assumption of uniform size distribution of particles in a solution. Our data and the literature^10^, ^39^ suggest that there are two distinct particle species/sizes, as a result of the composite composition of the mixed micelles. Therefore, data should be interpreted with caution. Despite this limitation, DLS is increasingly used for *in vitro* research and in publications characterizing mixed micelles because of its advantage of being a high throughput, low cost and technically accessible method for the assessment under near‐native conditions.

Although the software used in the present study applies a calculation adjusted for polydisperse size distributions (non‐negative least squares, NNLS),^40^ the drawback of NNLS is its sensitivity to small variations in measurements,^23^ The constrained regularization method for inverting data (CONTIN) calculation serves as a modified NNLS method resulting in narrower size distribution by reducing scattering noise of intensity data^23^ and can be used as an alternative for polydisperse and noisy data, if the size is the major outcome parameter.

### Recommendations on particle size report

When considering DSL data, it is important to consider that intensity, volume and number distributions are different representations of the same physical reality of the size distribution and only approximations of the true particle size distribution. The mixed micellar fractions obtained from *in vitro* digestion were largely polydisperse, with at least two or three peaks, limiting the single use of the mean particle diameter (*z*‐average) to describe size characteristics. For the particle size characterization of polydisperse solutions from *in vitro* digestions, the following is suggested: first, the PDI, intensity and volume distributions should be considered. For monodispersed samples [low PDI (< 0.2) and similar intensity and volume distributions], only reporting the mean particle diameter, is sufficient. However, for the more often encountered polydisperse samples, the volume distribution curve or individual volume‐weighted peak sizes should be reported. For a better understanding of the number‐to‐volume relationship, the number distribution as well as number mean should also be included. To improve the accuracy of the transformation of volume data from the scattering intensity data, the correct refractive index and absorbance of the sample (e.g. at 633 nm for Zetasizer® ZSP) should be used for data derived from the intensity distribution. We used olive oil in not emulsified form for the *in vitro* digestions. To expand the recommendation given in this work to mixed micelles with varying composition, subsequent studies should implement additional oils differing in carbon chain length and saturation of fatty acids. Emulsification can be implemented as additional factor because the droplet size can have an influence in mixed micelles size.^9^, ^10^ Our work reflects more natural/unmodified digestion conditions of the oil compared to emulsification of the oil prior to *in vitro* digestion.

### Effect of storage on mixed micellar size and surface charge

The reduction of the majority the smaller particles (< 100 nm) and the substantial increase in larger particles above the bioaccessibility filter cut‐off (< 200 nm) suggests that freezing of the filtered fraction promotes irreversible aggregation or sedimentation of particles in the mixed micellar fraction (Fig. 2A). It has been reported that the size of mixed micelles is temperature‐dependent,^34^, ^37^, ^41^ and a temperature decrease from 60 to 20 °C increased the hydrodynamic radius from 30 to 60 nm, depending on the bile salts used.^37^ A possible reason for the temperature‐dependence of mixed micellar fraction is the changed structure of water leading to altered water‐bile salt interactions.^42^

The heterogeneity of the mixed micellar fraction increased significantly after freezing (Fig. 1; see also Supporting information, Table S2). The increased heterogeneity was associated with a broader volume‐weighted distribution and the presence of additional larger particles (probably aggregates^37^, ^43^) (Fig. 2A, dark grey graph).

Although the volume‐weighted representation indicated larger particle sizes upon freezing, the number distribution and number mean were negligibly influenced by the freezing, if at all (Fig. 2B; see also Supporting information, Table S8). We observed changes of particle distribution towards bigger particles, but simultaneously the number distribution did not change upon freezing. It may thus be concluded that the presence of big particles may be attributed to a relatively small amount of large aggregates, and that most mixed micelles did not change particle size notably. Further studies on the effect of freezing should assess whether the absolute amount of particles is affected by freezing to complement this finding.

Our findings are in agreement with other studies reporting a similar particle diameter of the *in vitro* mixed micellar fraction.^10^, ^39^, ^44^ It has been reported that the mixed micellar fraction from *in vitro* digestion experiments consists of two distinct particle sizes.^10^, ^39^, ^45^ The smaller particles were in the range 30–70 nm and the bigger particles were in the range 90–210 nm.^39^ It was assumed that the smaller particles represented the mixed micelles, whereas the bigger fraction consist of phospholipid vesicles.^39^

The mean pore size of intestinal mucus (200 nm) limited the diffusion of large (500 nm) compared to small particles (100 nm),^46^ suggesting a size‐dependent absorption of nanoparticles. When a higher proportion of aggregates bigger than the mucus pore size (> 200 nm) is observed in the micellar fraction, we assume that the amount of micelles in solution carrying bioactives is reduced and by it the amount of absorbable (bioaccessible) proportion leading to lower bioaccessibility. Together with the observations that bigger particles (> 200 nm) are less absorbed in the intestine,^46^, ^47^, ^48^ a higher proportion of bigger particles could serve as an indicator of lower bioaccessibility.

It cannot be excluded that other food matrix or digestive compounds, such as the digestive enzymes, contributed in part to the measured size distribution. The major enzymes used in *in vitro* digestions (pepsin, lipase, trypsin), have a size range of approximately 1.1–6.6 nm,^49^, ^50^, ^51^ which makes it possible that these enzymes could have been measured in the first peak at 10 nm. However, when looking at empty digestion (= no addition of compounds or food), no size peak of 10 nm was detected, supporting the notion that the 10 nm peak in mixed micelle samples originates from the compounds digested rather than the digestive enzymes (see Supporting information, Fig. S8). However, it is possible that the enzymes agglomerated into bigger particles or attached to the micellar structures affecting the measured size and charge.^52^ Indeed, the size of the nanoparticles was reported to be higher when incubated with digestive enzymes forming a protein corona on the surface of nanoparticles.^52^

*ζ*‐potential, expressed in millivolts (mV), is the most common way to report surface charge of particles in a colloidal solution. The *ζ*‐potential indicates the charge at the slipping plane of a moving colloid particle.^53^ All samples had a negative surface charge as a result of the negative charge of bile salts under physiological conditions,^45^ and free fatty acids, released from olive oil during *in vitro* digestion. *ζ*‐potential values showed a narrow distribution and were not affected by freezing emphasizing the stability and reproducibility of this parameter. The surface charge of digested compounds and foods was comparable to empty digestion, whereas oil decreased the surface charge, probably because of the generation of negatively charged free fatty acids by lipases during *in vitro* digestion. Hence, surface charge is more affected by the addition of oil, than the type of compound digested in this work.

## CONCLUSIONS

The volume‐weighted size distribution indicated two main particle sizes with high amount of particles above the filter cut‐off (< 200 nm) suggesting mixed micellar fraction undergo dynamic size changes. The polydispersity was high (PDI > 0.3) for almost half of the mixed micellar fractions of the total dataset. Based on the presented data, we recommend the use of volume‐weighted size distribution curves, in addition to the polydispersity index and mean particle diameter data, when aiming to characterize the size of particles in the predominantly polydisperse mixed micellar fractions from *in vitro* digestion experiments. Because freezing the mixed micellar fraction led to particle aggregation, whereas freezing of the unfiltered digesta reduced freezing‐induced aggregation, we further recommend freezing of the latter if particle size measurements cannot be performed on the day of *in vitro* digestion and filtering of the thawed digesta prior to analyses. Importantly, this freezing‐induced aggregation (size shift) should also be considered and avoided in subsequent experiments (e.g. cellular uptake) where the size and/or stability of the micelles might affect the outcome. *ζ*‐potential was not affected by sample freezing, and thus is considered a stable and reproducible parameter for the surface charge of mixed micellar fractions.

## CONFLICTS OF INTEREST

The authors declare that they have no conflicts of interest.

## Supporting information


**Figure S1.** Relationship between intensity‐, volume‐ and number‐weighted size distribution, and the complementary data calculated from these used to report the size characteristics of *in vitro* mixed micellar fraction after digestion of vitamin E. (A) Scattering intensity distribution is the primary DLS data, from which the (B) volume‐ and (C) number‐weighted distribution is derived. Volume and number distributions represent the particle volume or number of particles, at a certain size (nm), as a percentage of the total volume or total number of particles in the sample. Intensity distribution can be summarized as (A) mean particle diameter (*z*‐average, nm). Volume‐weighted particle distribution can be summarized as (B) mean particle size of individual peaks and the percentage area under the curve (AUC) of each peak relative to the total AUC. Number‐weighted particle distribution can be summarized as (C) number mean (nm). Size distribution data are depicted as means on a logarithmic scale (*n* ≥ 8).

**Figure S2.** Size distributions of the *in vitro* mixed micellar fraction according to (A) intensity‐weighted, (B) volume‐weighted^†^ and (B) number‐weighted^†^ size distributions of the *in vitro* mixed micellar fraction, depending on the dilution medium used for the size measurement. Samples were measured directly after digestion (filtered), after storage (freezing) (filtered‐frozen) or after storage (freezing) of the unfiltered fraction, followed by filtration directly before the measurement (frozen‐filtered). The mixed micellar fraction of foods digested with or without olive oil were diluted 1:10 in H_2_Odd or simulated intestinal fluid (SIF). Data are depicted as means of all treatments (filtered, filtered‐frozen, frozen‐filtered) on a logarithmic scale (*n* = 15). ^†^Volume or number of particles at a specific size, as percentage of the total volume or number of particles, respectively.

**Figure S3.** Surface charge (*ζ*‐potential) of particles in the mixed micellar fraction measured directly after *in vitro* digestion (filtered) of individual compounds with or without olive oil, after freezing (filtered‐frozen) or after freezing the unfiltered fraction, followed by filtration (frozen‐filtered). Data are depicted as the mean ± SD (*n* = 12).

**Figure S4.** Surface charge (*ζ*‐potential) of particles in the mixed micellar fraction measured directly after *in vitro* digestion (filtered) of spinach and red cabbage with or without olive oil, after freezing (filtered‐frozen) or after freezing the unfiltered fraction, followed by filtration (frozen‐filtered). (A) Simplified *in vitro* digestion according to Rodrigues *et al*. (1) and the (B) standardized *in vitro* digestion according to Brodkorb *et al*. (2) were performed. Data are depicted as the mean ± SD (*n* = 12).

**Figure S5.** The effects of different combinations of storage (freezing) and filtering (200 nm) on (A) the volume‐weighted^†^ and (B) number‐weighted^†^ size distributions of the *in vitro* mixed micellar fraction measured in SIF. Samples were measured directly after digestion (filtered), after storage (freezing) (filtered‐frozen) or after storage (freezing) of the unfiltered fraction, followed by filtration directly before the measurement (frozen‐filtered). Distributions represent the mean (*n* = 5) of all *in vitro* digestions performed with food, with or without addition of olive oil (*n* ≥ 6) and are depicted on a logarithmic scale. ^†^Volume or number of particles at a specific size, as percentage of the total volume or number of particles, respectively.

**Figure S6.** The effect of storage on the surface charge (*ζ*‐potential) of particles in the mixed micellar fraction. Results of the *in vitro* mixed micellar fraction measured directly after in vitro digestion (^†^filtered) are compared to the same sample (line connections) measured after storage (freezing) (^‡^filtered‐frozen) and compared to the same sample of which the unfiltered fraction was stored (frozen), followed by filtration directly before the measurement (^§^frozen‐filtered). Each graph shows all *in vitro* digestions performed (*n* = 24). Each dot represents the mean of one sample (*n* = 3).

**Figure S7.** Comparison of the intensity, volume‐ and number‐weighted distributions of a hypothetical solution containing equal numbers of 5 nm and 50 nm spherical particles. Adapted from the Zetasizer® Nano ZSP manual.

**Figure S8.** Volume‐weighted size distributions (nm) of the *in vitro* mixed micellar fraction of the control digestion. For the control digestion, compounds and food was replaced by 1 mL of deionized H_2_O. Control digestions had similar enzyme activities, electrolyte concentration and incubation times as digested individual compounds and food samples. Volume‐weighted size distributions representing the volume of particles at a specific size, as percentage of the total volume of particles, respectively. Data are depicted as means on a logarithmic scale (*n* = 10).

**Table S1.** Digestive parameters of the in vitro digestion protocol from Rodrigues et al.^1^ and Brodkorb et al.^2^

**Table S2.** The effects of different combinations of storage (freezing) and filtering (200 nm) on the polydispersity index (PDI) of in vitro mixed micellar fractions measured directly after digestion (**filtered**), after freezing (**filtered‐frozen**), or after freezing the unfiltered fraction, followed by filtration (**frozen‐filtered**).

**Table S3.** Percentage of particles (volume‐weighted), in the in vitro mixed micellar fraction, bigger than the filter cut‐off of 200 nm.

**Table S4.** Raw size distribution data (nm) of particles in the in vitro mixed micellar fraction of vitamin E. Size is represented as volume‐weighted distribution, which is the volume of particles at a specific size, as percentage of the total volume of particles. Three separate in vitro digestions were performed (#1‐3) and each measured by DLS in four consecutive runs (1‐4).

**Table S5.** The effects of different combinations of storage (freezing) and filtering (200 nm) on the polydispersity index (PDI), the z‐average (mean particle diameter of intensity size distribution), and the number mean (mean particle size of number‐weighted size distribution) of in vitro mixed micellar fractions diluted in simulated intestinal fluid (SIF) directly after digestion (**filtered**), after freezing (**filtered‐frozen**), or after freezing the unfiltered fraction, followed by filtration (**frozen‐filtered**).

**Table S6.** Raw size distribution data (nm) of particles in the in vitro mixed micellar fraction of spinach diluted in simulated intestinal fluid (SIF). Size is represented as volume‐weighted distribution, which is the volume of particles at a specific size, as percentage of the total volume of particles. Three separate in vitro digestions were performed (#1‐3) and each measured by DLS in three consecutive runs (1‐3).

**Table S7.** The effects of different combinations of storage (freezing) and filtering (200 nm) on the z‐average (mean particle diameter of intensity size distribution) of in vitro mixed micellar fractions measured directly after digestion (**filtered**), after freezing (**filtered‐frozen**), or after freezing the unfiltered fraction, followed by filtration (**frozen‐filtered**).

**Table S8.** The effects of different combinations of storage (freezing) and filtering (200 nm) on the number mean (mean particle size of number‐weighted size distribution) of in vitro mixed micellar fractions measured directly after digestion (**filtered**), after freezing (**filtered‐frozen**), or after freezing the unfiltered fraction, followed by filtration (**frozen‐filtered**).

## Data Availability

The data that support the findings of this study are available from the corresponding author upon reasonable request.
