# Supplementary material for: High heterogeneity in the size distribution of the micellar fraction from in vitro digestions: sample preparation and reporting recommendations
Source: J Sci Food Agric. 2025 Jan 7;105(6):3406–15. doi: 10.1002/jsfa.14109 (PMC11949856; doi:10.1002/jsfa.14109)
Supplement: Supplementary file 1 — Figure S1. Relationship between intensity‐, volume‐ and number‐weighted size distribution, and the complementary data calculated from these used to report the size characteristics of in vitro mixed micellar fraction after digestion of vitamin E. (A) Scattering intensity distribution is the primary DLS data, from which the (B) volume‐ and (C) number‐weighted distribution is derived. Volume and number distributions represent the particle volume or number of particles, at a certain size (nm), as a percentage of the total volume or total number of particles in the sample. Intensity distribution can be summarized as (A) mean particle diameter (z‐average, nm). Volume‐weighted particle distribution can be summarized as (B) mean particle size of individual peaks and the percentage area under the curve (AUC) of each peak relative to the total AUC. Number‐weighted particle distribution can be summarized as (C) number mean (nm). Size distribution data are depicted as means on a logarithmic scale (n ≥ 8). [file JSFA-105-3406-s001.docx]

**Figure S1** Relationship between intensity-, volume-, and number-weighted size distribution, and the complementary data calculated from these used to report the size characteristics of in vitro mixed micellar fraction after digestion of vitamin E. (**A**) Scattering intensity distribution is the primary DLS data, from which the (**B**) volume- and (**C**) number-weighted distribution is derived. Volume and number distributions represent the particle volume or number of particles, at a certain size (nm), as percentage of the total volume or total number of particles in the sample. Intensity distribution can be summarised as (**A**) mean particle diameter (z-average, nm). Volume-weighted particle distribution can be summarised as (**B**) mean particle size of individual peaks and the percentage area under the curve (AUC) of each peak relative to the total AUC. Number-weighted particle distribution can be summarised as (**C**) number mean (nm). Size distribution data are depicted as means on a logarithmic scale (n ≥ 8).
