# Supplementary material for: High heterogeneity in the size distribution of the micellar fraction from in vitro digestions: sample preparation and reporting recommendations
Source: J Sci Food Agric. 2025 Jan 7;105(6):3406–15. doi: 10.1002/jsfa.14109 (PMC11949856; doi:10.1002/jsfa.14109)
Supplement: Supplementary file 2 — Figure S2. Size distributions of the in vitro mixed micellar fraction according to (A) intensity‐weighted, (B) volume‐weighted† and (B) number‐weighted† size distributions of the in vitro mixed micellar fraction, depending on the dilution medium used for the size measurement. Samples were measured directly after digestion (filtered), after storage (freezing) (filtered‐frozen) or after storage (freezing) of the unfiltered fraction, followed by filtration directly before the measurement (frozen‐filtered). The mixed micellar fraction of foods digested with or without olive oil were diluted 1:10 in H2Odd or simulated intestinal fluid (SIF). Data are depicted as means of all treatments (filtered, filtered‐frozen, frozen‐filtered) on a logarithmic scale (n = 15). †Volume or number of particles at a specific size, as percentage of the total volume or number of particles, respectively. [file JSFA-105-3406-s009.docx]

**Figure S2** Size distributions of the *in vitro* mixed micellar fraction according to (**A**) intensity-weighted, (**B**) volume-weighted^†^ and (**B**) number-weighted^†^ size distributions of the *in vitro* mixed micellar fraction, depending on the dilution medium used for the size measurement. Samples were measured directly after digestion (**filtered**), after storage (**freezing**) (filtered-frozen) or after storage (**freezing**) of the unfiltered fraction, followed by filtration directly before the measurement (frozen-filtered). The mixed micellar fraction of foods digested with or without olive oil were diluted 1:10 in H_2_Odd or simulated intestinal fluid (SIF). Data are depicted as means of all treatments (**filtered, filtered-frozen, frozen-filtered**) on a logarithmic scale (n = 15). ^†^– volume or number of particles at a specific size, as percentage of the total volume or number of particles, respectively.
