# Supplementary material for: High heterogeneity in the size distribution of the micellar fraction from in vitro digestions: sample preparation and reporting recommendations
Source: J Sci Food Agric. 2025 Jan 7;105(6):3406–15. doi: 10.1002/jsfa.14109 (PMC11949856; doi:10.1002/jsfa.14109)
Supplement: Supplementary file 3 — Figure S3. Surface charge (ζ‐potential) of particles in the mixed micellar fraction measured directly after in vitro digestion (filtered) of individual compounds with or without olive oil, after freezing (filtered‐frozen) or after freezing the unfiltered fraction, followed by filtration (frozen‐filtered). Data are depicted as the mean ± SD (n = 12). [file JSFA-105-3406-s011.docx]

**Figure S3** Surface charge (ζ-potential) of particles in the mixed micellar fraction measured directly after *in vitro* digestion (**filtered**) of individual compounds with or without olive oil, after freezing (**filtered-frozen**) or after freezing the unfiltered fraction, followed by filtration (**frozen-filtered**). Data are depicted as mean ± SD (n=12).
