# Supplementary material for: High heterogeneity in the size distribution of the micellar fraction from in vitro digestions: sample preparation and reporting recommendations
Source: J Sci Food Agric. 2025 Jan 7;105(6):3406–15. doi: 10.1002/jsfa.14109 (PMC11949856; doi:10.1002/jsfa.14109)
Supplement: Supplementary file 4 — Figure S4. Surface charge (ζ‐potential) of particles in the mixed micellar fraction measured directly after in vitro digestion (filtered) of spinach and red cabbage with or without olive oil, after freezing (filtered‐frozen) or after freezing the unfiltered fraction, followed by filtration (frozen‐filtered). (A) Simplified in vitro digestion according to Rodrigues et al. (1) and the (B) standardized in vitro digestion according to Brodkorb et al. (2) were performed. Data are depicted as the mean ± SD (n = 12). [file JSFA-105-3406-s002.docx]

**Figure S4** Surface charge (ζ-potential) of particles in the mixed micellar fraction measured directly after *in vitro* digestion (**filtered**) of spinach and red cabbage with or without olive oil, after freezing (**filtered-frozen**) or after freezing the unfiltered fraction, followed by filtration (**frozen-filtered**). (**A**) Simplified *in vitro* digestion according to Rodrigues et al. (1) and the (**B**) standardized *in vitro* digestion according to Brodkorb et al. (2) were performed. Data are depicted as mean ± SD (n=12).

Literature Cited

1. Rodrigues DB, Mariutti LRB, Mercadante AZ. An in vitro digestion method adapted for carotenoids and carotenoid esters: moving forward towards standardization. Food Funct 2016; 7(12):4992–5001.

2. Brodkorb A, Egger L, Alminger M, Alvito P, Assunção R, Ballance S et al. INFOGEST static in vitro simulation of gastrointestinal food digestion. Nat Protoc 2019; 14(4):991–1014.
