# Supplementary material for: High heterogeneity in the size distribution of the micellar fraction from in vitro digestions: sample preparation and reporting recommendations
Source: J Sci Food Agric. 2025 Jan 7;105(6):3406–15. doi: 10.1002/jsfa.14109 (PMC11949856; doi:10.1002/jsfa.14109)
Supplement: Supplementary file 5 — Figure S5. The effects of different combinations of storage (freezing) and filtering (200 nm) on (A) the volume‐weighted† and (B) number‐weighted† size distributions of the in vitro mixed micellar fraction measured in SIF. Samples were measured directly after digestion (filtered), after storage (freezing) (filtered‐frozen) or after storage (freezing) of the unfiltered fraction, followed by filtration directly before the measurement (frozen‐filtered). Distributions represent the mean (n = 5) of all in vitro digestions performed with food, with or without addition of olive oil (n ≥ 6) and are depicted on a logarithmic scale. †Volume or number of particles at a specific size, as percentage of the total volume or number of particles, respectively. [file JSFA-105-3406-s016.docx]

**Figure S5** The effects of different combinations of storage (freezing) and filtering (200 nm) on (**A**) the volume-weighted^†^ and (**B**) number-weighted^†^ size distributions of the in vitro mixed micellar fraction measured in SIF. Samples were measured directly after digestion (**filtered**), after storage (freezing) (**filtered-frozen**) or after storage (freezing) of the unfiltered fraction, followed by filtration directly before the measurement (**frozen-filtered**). ^†^ – volume or number of particles at a specific size, as percentage of the total volume or number of particles, respectively. Distributions represent the mean (n=5) of all in vitro digestions performed with food, with or without addition of olive oil (n ≥ 6) and are depicted on a logarithmic scale.
