# Supplementary material for: High heterogeneity in the size distribution of the micellar fraction from in vitro digestions: sample preparation and reporting recommendations
Source: J Sci Food Agric. 2025 Jan 7;105(6):3406–15. doi: 10.1002/jsfa.14109 (PMC11949856; doi:10.1002/jsfa.14109)
Supplement: Supplementary file 6 — Figure S6. The effect of storage on the surface charge (ζ‐potential) of particles in the mixed micellar fraction. Results of the in vitro mixed micellar fraction measured directly after in vitro digestion (†filtered) are compared to the same sample (line connections) measured after storage (freezing) (‡filtered‐frozen) and compared to the same sample of which the unfiltered fraction was stored (frozen), followed by filtration directly before the measurement (§frozen‐filtered). Each graph shows all in vitro digestions performed (n = 24). Each dot represents the mean of one sample (n = 3). [file JSFA-105-3406-s007.docx]

**Figure S6** The effect of storage on the surface charge (ζ-potential) of particles in the mixed micellar fraction. Results of the in vitro mixed micellar fraction measured directly after in vitro digestion ((^†^**filtered**) are compared to the same sample (line connections) measured after storage (freezing) (^‡^**filtered-frozen**) and compared to the same sample of which the unfiltered fraction was stored (frozen), followed by filtration directly before the measurement (^§^**frozen-filtered**). Each graph shows all in vitro digestions performed (n=24). Each dot represents the mean of one sample (n=3).
