# Supplementary material for: High heterogeneity in the size distribution of the micellar fraction from in vitro digestions: sample preparation and reporting recommendations
Source: J Sci Food Agric. 2025 Jan 7;105(6):3406–15. doi: 10.1002/jsfa.14109 (PMC11949856; doi:10.1002/jsfa.14109)
Supplement: Supplementary file 7 — Figure S7. Comparison of the intensity, volume‐ and number‐weighted distributions of a hypothetical solution containing equal numbers of 5 nm and 50 nm spherical particles. Adapted from the Zetasizer® Nano ZSP manual. [file JSFA-105-3406-s012.docx]

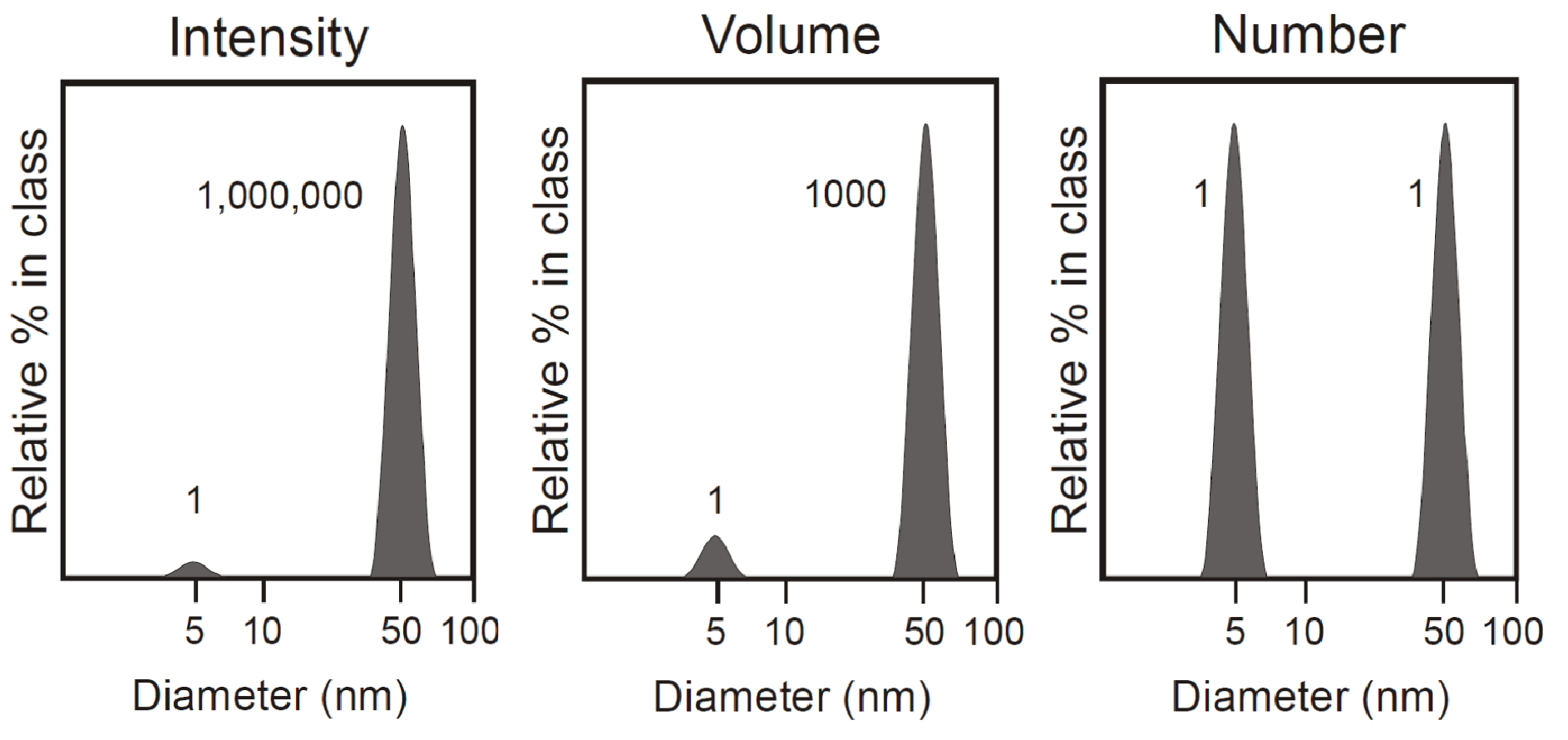


**Figure S7** Comparison of the intensity, volume- and number-weighted distributions of a hypothetical solution containing equal numbers of 5 nm and 50 nm spherical particles. Adapted from Zetasizer® Nano ZSP manual.
