# Supplementary material for: High heterogeneity in the size distribution of the micellar fraction from in vitro digestions: sample preparation and reporting recommendations
Source: J Sci Food Agric. 2025 Jan 7;105(6):3406–15. doi: 10.1002/jsfa.14109 (PMC11949856; doi:10.1002/jsfa.14109)
Supplement: Supplementary file 8 — Figure S8. Volume‐weighted size distributions (nm) of the in vitro mixed micellar fraction of the control digestion. For the control digestion, compounds and food was replaced by 1 mL of deionized H2O. Control digestions had similar enzyme activities, electrolyte concentration and incubation times as digested individual compounds and food samples. Volume‐weighted size distributions representing the volume of particles at a specific size, as percentage of the total volume of particles, respectively. Data are depicted as means on a logarithmic scale (n = 10). [file JSFA-105-3406-s004.docx]

**Figure S8** Volume-weighted size distributions (nm) of the *in vitro* mixed micellar fraction of the control digestion. For the control digestion, compounds and food was replaced by 1mL H20dd. Control digestions had similar enzyme activities, electrolyte concentration and incubation times as digested individual compounds and food samples. Volume-weighted size distributions representing the volume of particles at a specific size, as percentage of the total volume of particles, respectively. Data are depicted as means on a logarithmic scale (n = 10).
