# Supplementary material for: High heterogeneity in the size distribution of the micellar fraction from in vitro digestions: sample preparation and reporting recommendations
Source: J Sci Food Agric. 2025 Jan 7;105(6):3406–15. doi: 10.1002/jsfa.14109 (PMC11949856; doi:10.1002/jsfa.14109)
Supplement: Supplementary file 9 — Table S1. Digestive parameters of the in vitro digestion protocol from Rodrigues et al. 1 and Brodkorb et al. 2 [file JSFA-105-3406-s005.docx]

**Table S1** Digestive parameters of the *in vitro* digestion protocol from Rodrigues et al.^1^ and Brodkorb et al.^2^

|  | **Simplified Method  (Rodrigues et al.)** | **INFOGEST 2.0 Method  (Brodkorb et al.)** |
| --- | --- | --- |
|  | **Incubation temperature: 37 °C Shaking water bath: 180 rpm** | |
| **Oral phase** |  |  |
| Duration | - | 5 min |
| pH | - | not adjusted  (pH of SSF = 7.0) |
| Solutions | - | 2 mL simulated salivary fluid (SSF) |
| Enzymes (U mL^-1^) ^†^ | - | - |
| Enzymes (mg mL^-1^) ^†^ | - | - |
| Final volume | - | 3 mL |
|  |  |  |
| **Gastric phase** |  |  |
| Duration | 1 h | 2 h |
| pH | 2.5 ± 0.1 | 2.5 ± 0.1 |
| Solutions | 8.55 mL 0.9 % NaCl | 4 mL simulated gastric fluid (SGF) |
| Enzymes (U mL^-1^) ^†^ | Pepsin (label ≥ 250 U mg^-1^)  in 0.1 M HCl | Pepsin: 2346 (in-lab = 782 U mg^-1^) |
| Enzymes (mg mL^-1^) ^†^ | 4.9 | 3 |
| Final volume | 10 mL | 7 mL |
|  |  |  |
| **Intestinal phase** |  |  |
| Duration | 2 h | 2 h |
| pH | 6.5 ± 0.1 | 7.0 ± 0.1 |
| Solutions | 3.5 mL porcine bile in 0.1 M NaHCO3 (31 mg mL^-1^) | 2 mL bovine bile in simulated intestinal fluid (SIF) (35.4 mg mL^-1^) 1.6 mL H_2_Odd |
| Enzymes (U mL^-1^) ^†^ | Pancreatin (labeled: 8 × USP specifications) Lipase (labeled: 30-90 U mg^-1^ protein using triacetin as substrate) | Pancreatin: 1667  (in-lab lipase activity = 72 U mg^-1^) Lipase: 465  (in-lab lipase activity = 95 U mg^-1^) Total Lipase activity: 2129 |
| Enzymes (mg mL^-1^) ^†^ | Pancreatin 1.3 Lipase 0.6 | Pancreatin 23.1 Lipase 4.9 |
| Final volume | 14 mL | 15 mL |
| ^†^expressed per final volume of the respective digestive phase | | |
