# Supplementary material for: High heterogeneity in the size distribution of the micellar fraction from in vitro digestions: sample preparation and reporting recommendations
Source: J Sci Food Agric. 2025 Jan 7;105(6):3406–15. doi: 10.1002/jsfa.14109 (PMC11949856; doi:10.1002/jsfa.14109)
Supplement: Supplementary file 11 — Table S3. Percentage of particles (volume‐weighted), in the in vitro mixed micellar fraction, bigger than the filter cut‐off of 200 nm. [file JSFA-105-3406-s008.docx]

**Table S3** Percentage of particles (volume-weighted), in the *in vitro* mixed micellar fraction, bigger than the filter cut‑off of 200 nm

|  | **Particles >200nm [volume %]** | | |
| --- | --- | --- | --- |
|  | **Filtered** | **Filtered-Frozen** | **Frozen-Filtered** |
| *Pure compounds (simplified digestion)* |  |  |  |
| **Vitamin E** | 60.0 | 82.5 | 61.8 |
| **Vitamin E + olive oil** | 47.4 | 43.2 | 43.1 |
| **Vitamin A** | 17.6 | 61.4 | 25.2 |
| **Vitamin A + olive oil** | 19.7 | 61.4 | 29.8 |
| **β-carotene** | 29.4 | 63.8 | 26.5 |
| **β-carotene + olive oil** | 28.5 | 39.9 | 16.0 |
| **Curcumin** | 40.5 | 68.0 | 24.7 |
| **Curcumin + olive oil** | 28.1 | 46.6 | 20.5 |
| **Naringenin** | 35.8 | 84.1 | 40.8 |
| **Naringenin + olive oil** | 21.2 | 19.9 | 28.7 |
| **Compound mix** | 16.2 | 85.7 | 28.2 |
| **Compound mix + olive oil** | 18.2 | 19.5 | 21.9 |
| **Olive oil** | 31.8 | 57.7 | 12.7 |
| **Control (empty digestion)** | 50.7 | 56.5 | 28.9 |
|  |  |  |  |
| *Foods (simplified digestion)* |  |  |  |
| **Spinach** | 1.0 | 5.4 | 2.0 |
| **Spinach + olive oil** | 18.6 | 83.7 | 43.3 |
| **Red cabbage** | 2.7 | 47.4 | 40.5 |
| **Red cabbage + olive oil** | 9.2 | 46.1 | 26.9 |
| **Control (empty digestion)** | 30.6 | 77.9 | 43.1 |
|  |  |  |  |
| *Foods (INFOGEST 2.0)* |  |  |  |
| **Spinach** | 40.8 | 28.2 | 19.1 |
| **Spinach + olive oil** | 12.6 | 50.1 | 43.8 |
| **Red cabbage** | 8.7 | 47.6 | 13.8 |
| **Red cabbage + olive oil** | 11.1 | 38.9 | 17.7 |
| **Control (empty digestion)** | 22.8 | 62.1 | 25.2 |

Samples were measured directly after *in vitro* digestion (**filtered**), after freezing (**filtered-frozen**), or after freezing the unfiltered fraction, followed by filtration (**frozen-filtered**). Data are represented as mean (n ≥ 8)
