# Supplementary material for: High heterogeneity in the size distribution of the micellar fraction from in vitro digestions: sample preparation and reporting recommendations
Source: J Sci Food Agric. 2025 Jan 7;105(6):3406–15. doi: 10.1002/jsfa.14109 (PMC11949856; doi:10.1002/jsfa.14109)
Supplement: Supplementary file 13 — Table S5. The effects of different combinations of storage (freezing) and filtering (200 nm) on the polydispersity index (PDI), the z‐average (mean particle diameter of intensity size distribution), and the number mean (mean particle size of number‐weighted size distribution) of in vitro mixed micellar fractions diluted in simulated intestinal fluid (SIF) directly after digestion (filtered), after freezing (filtered‐frozen), or after freezing the unfiltered fraction, followed by filtration (frozen‐filtered). [file JSFA-105-3406-s006.docx]

**Table S5** The effects of different combinations of storage (freezing) and filtering (200 nm) on the polydispersity index (PDI), the z-average (mean particle diameter of intensity size distribution), and the number mean (mean particle size of number-weighted size distribution) of in vitro mixed micellar fractions diluted in simulated intestinal fluid (SIF) directly after digestion (**filtered**), after freezing (**filtered-frozen**), or after freezing the unfiltered fraction, followed by filtration (**frozen-filtered**)

|  | **Micellar Fraction measured in SIF** | | |
| --- | --- | --- | --- |
| **Treatment** | **Filtered** | **Filtered-Frozen** | **Frozen-Filtered** |
|  |  |  |  |
| *Food (INFOGEST 2.0)* | **PDI** | | |
|  |  |  |  |
| **Spinach** | 0.306 ± 0.029 | 0.232 ± 0.013 * | 0.254 ± 0.021 |
| **Spinach + olive oil** | 0.513 ± 0.048 | 0.813 ± 0.098 * | 0.509 ± 0.030 |
| **Red cabbage** | 0.229 ± 0.008 | 0.357 ± 0.042 * | 0.316 ± 0.022 * |
| **Red cabbage + olive oil** | 0.360 ± 0.114 | 0.542 ± 0.031 * | 0.439 ± 0.024 * |
| **Control (empty digestion)** | 0.307 ± 0.045 | 0.271 ± 0.007 | 0.297 ± 0.048 |
|  |  |  |  |
|  | **Z-Average [nm]** | | |
|  |  |  |  |
| **Spinach** | 235.2 ± 9.7 | 211.1 ± 2.7 | 226.5 ± 13.8 |
| **Spinach + olive oil** | 219.7 ± 7.5 | 710.9 ± 88.6 * | 107.1 ± 5.5 * |
| **Red cabbage** | 167.5 ± 6.2 | 176.8 ± 24.1 | 179.0 ± 21.9 |
| **Red cabbage + olive oil** | 200.0 ± 91.1 | 162.4 ± 38.4 | 104.6 ± 3.8 * |
| **Control (empty digestion)** | 163.2 ± 4.6 | 154.0 ± 5.8 | 175.8 ± 11.6 |
|  |  |  |  |
|  | **Number mean [nm]** | | |
|  |  |  |  |
| **Spinach** | 101.4 ± 52.2 | 140.8 ± 50.9 | 138.4 ± 46.1 |
| **Spinach + olive oil** | 15.4 ± 1.8 | 70.0 ± 46.9 * | 14.6 ± 3.1 |
| **Red cabbage** | 92.2 ± 24.6 | 74.2 ± 17.5 | 65.7 ± 29.2 |
| **Red cabbage + olive oil** | 96.0 ± 113.3 | 19.3 ± 6.9 * | 16.3 ± 6.4 * |
| **Control (empty digestion)** | 60.5 ± 31.5 | 97.5 ± 16.3 | 100.8 ± 22.4 |

Values are given as mean ± SD (n ≥ 6). Asterisk indicates significant difference (p<0.05) compared to the “**filtered**” group
