# Supplementary material for: High heterogeneity in the size distribution of the micellar fraction from in vitro digestions: sample preparation and reporting recommendations
Source: J Sci Food Agric. 2025 Jan 7;105(6):3406–15. doi: 10.1002/jsfa.14109 (PMC11949856; doi:10.1002/jsfa.14109)
Supplement: Supplementary file 14 — Table S6. Raw size distribution data (nm) of particles in the in vitro mixed micellar fraction of spinach diluted in simulated intestinal fluid (SIF). Size is represented as volume‐weighted distribution, which is the volume of particles at a specific size, as percentage of the total volume of particles. Three separate in vitro digestions were performed (#1‐3) and each measured by DLS in three consecutive runs (1‐3). [file JSFA-105-3406-s003.docx]

**Table S6** Raw size distribution data (nm) of particles in the in vitro mixed micellar fraction of spinach diluted in simulated intestinal fluid (SIF). Size is represented as volume-weighted distribution, which is the volume of particles at a specific size, as percentage of the total volume of particles. Three separate in vitro digestions were performed (#1-3) and each measured by DLS in three consecutive runs (1-3).
