# Supplementary material for: High heterogeneity in the size distribution of the micellar fraction from in vitro digestions: sample preparation and reporting recommendations
Source: J Sci Food Agric. 2025 Jan 7;105(6):3406–15. doi: 10.1002/jsfa.14109 (PMC11949856; doi:10.1002/jsfa.14109)
Supplement: Supplementary file 16 — Table S8. The effects of different combinations of storage (freezing) and filtering (200 nm) on the number mean (mean particle size of number‐weighted size distribution) of in vitro mixed micellar fractions measured directly after digestion (filtered), after freezing (filtered‐frozen), or after freezing the unfiltered fraction, followed by filtration (frozen‐filtered). [file JSFA-105-3406-s010.docx]

**Table S8** The effects of different combinations of storage (freezing) and filtering (200 nm) on the number mean (mean particle size of number-weighted size distribution) of in vitro mixed micellar fractions measured directly after digestion (**filtered**), after freezing (**filtered-frozen**), or after freezing the unfiltered fraction, followed by filtration (**frozen-filtered**)

| **Number mean [nm]** | **Micellar Fraction** | | |
| --- | --- | --- | --- |
| **Treatment** | **Filtered** | **Filtered-Frozen** | **Frozen-Filtered** |
| *Pure compounds (simplified digestion)* |  |  |  |
| **Vitamin E** | 76.1±12.2 | 80.1±24.9 | 74.6±20.2 |
| **Vitamin E + olive oil** | 44.2±14.6 | 44.5± 6.2 | 50.0±25.6 |
| **Vitamin A** | 76.7± 5.9 | 80.2±15.0 | 76.5± 5.3 |
| **Vitamin A + olive oil** | 53.7± 4.8 | 48.6± 9.3 | 48.1±12.8 |
| **β-Carotene** | 78.8±10.3 | 77.2±16.3 | 80.1± 6.1 |
| **β-Carotene + olive oil** | 36.4± 5.3 | 39.9± 4.5 | 36.4±10.5 |
| **Curcumin** | 67.1± 8.2 | 85.8±10.2 * | 77.9± 8.9 |
| **Curcumin + olive oil** | 45.9±10.5 | 43.6±10.5 | 36.6±13.7 |
| **Naringenin** | 73.3± 7.9 | 81.4±21.4 | 70.8±15.0 |
| **Naringenin + olive oil** | 36.2± 4.7 | 44.6± 2.8 | 39.2± 4.6 |
| **Mix** | 79.8± 6.4 | 96.8±18.0 * | 72.7± 7.7 |
| **Mix + olive oil** | 36.2± 6.9 | 32.3± 4.1 | 45.7±11.4 |
| **Olive oil** | 49.8±13.3 | 33.7± 8.9 * | 31.9± 8.0 * |
| **Control (empty digestion)** | 80.2±13.4 | 79.4±20.0 | 80.8± 6.4 |
|  |  |  |  |
| *Food (Simplified digestion)* |  |  |  |
| **Spinach** | 9.7± 1.3 | 10.1± 0.7 | 9.3± 1.4 |
| **Spinach + olive oil** | 64.9±15.3 | 57.0±14.3 | 59.6±15.2 |
| **Red cabbage** | 10.0± 1.0 | 40.4±26.8 | 42.4±32.4 |
| **Red cabbage + olive oil** | 37.3±14.7 | 38.9±11.9 | 31.9±10.5 |
| **Control (empty digestion)** | 75.5± 7.3 | 84.9±15.2 | 71.0± 6.9 |
|  |  |  |  |
| *Food (INFOGEST 2.0)* |  |  |  |
| **Spinach** | 31.5±20.1 | 24.9±18.8 | 19.8±12.4 |
| **Spinach + olive oil** | 78.0± 6.4 | 39.2± 9.4 * | 57.0±23.5 |
| **Red cabbage** | 37.4±25.2 | 37.2±16.8 | 49.1±18.0 |
| **Red cabbage + olive oil** | 72.7± 7.5 | 33.7±13.7 * | 51.8±23.3 |
| **Control (empty digestion)** | 66.6±16.0 | 67.6±20.8 | 67.0±15.6 |

Values are given as mean ± SD (n ≥ 8). Asterisk indicates significant difference (p<0.05) compared to the “**filtered**” group.
